# The Changes in Size of Periapical Lesions after Root Canal Treatments Assessed by Digital Periapical Radiography and Cone-Beam Computed Tomography: A 2-Years Prospective Clinical Study

**DOI:** 10.3390/medicina58101437

**Published:** 2022-10-12

**Authors:** Jelena Gudac, Kristina Hellén-Halme, Vita Maciulskiene

**Affiliations:** 1 Clinic of Dental and Oral Pathology, Faculty of Odontology, Lithuanian University of Health Sciences, Eiveniu Str. 2, LT-50161 Kaunas, Lithuania; 2 Department of Oral and Maxillofacial Radiology, Faculty of Odontology, Malmö University, Carl Gustafs Väg 34, 21421 Malmö, Sweden

**Keywords:** apical periodontitis, cone-beam computed tomography, digital periapical radiography, root canal treatment outcome

## Abstract

*Background and Objectives:* There is limited information regarding comparison of long-term dynamics of periapical bone destruction estimated by digital periapical radiography (DPR) and by cone-beam computed tomography (CBCT). This study aimed to compare the radiographically assessed periapical changes of endodontically treated teeth over 2 years of follow-up and to analyse disagreements in periapical lesion size estimates around the same roots using DPR and CBCT. *Materials and Methods:* A total of 176 endodontically treated teeth, of 128 patients with apical periodontitis, were assessed by DPR and CBCT, at baseline and after 2 years. All periapical radiolucencies were categorised by severity (S0, S1, S2, S3) concerning their size. Descriptive statistics were used to report distribution of the radiolucencies at baseline and at follow-up, and their size transitions over 2 years. Site-specific comparison of the radiolucencies identified by two methods was performed using Z test and Pearson’s chi-square test. *Results:* majority of the detected radiolucencies were scored as S0: 65% and 68% at baseline; 89% and 83% at follow-up, by DPR and CBCT, respectively. Site-specific score comparison showed that disagreements comprised 18% and 20% of the total number of radiolucencies detected by DPR and CBCT, respectively. There were more disagreements between DPR and CBCT within categories S1 and S2 + S3 compared to S0: at baseline, they comprised 17–33% and after two years 62–95% of all detected radiolucencies within the category. 65% of non-matching score transitions over two years occurred between S0 and S1. The CBCT-based evaluation resulted in negative treatment outcomes for 10 more root canals than the DPR-based result. *Conclusions:* Most remarkable disagreement between DPR and CBCT recordings was observed within the radiolucency categories S2 and S3. However, the diagnostic accuracy of both radiographic methods was questionable as it resulted in a high proportion of non-matching S0-S1 lesion transitions over 2 years.

## 1. Introduction

Radiography is an essential diagnostic tool in endodontics, from disease diagnosis to long-term evaluation of healing outcomes. In combination with clinical findings, the treatment outcome is usually estimated by comparing pre-and postoperative radiographs [1]. Along with the conventional digital periapical radiography (DPR), the increasing application of cone-beam computed tomography (CBCT) has been acknowledged [2,3].

Traditionally, the DPR images have been used to evaluate the root canal morphology and the periapical structures for diagnosis and treatment planning and follow-up. However, this radiographic method has inherent limitations such as 2-dimensional diagnostic yield, superimposition and distortion of the essential structures that may hide lesions [4].

The 3-dimensional nature of CBCT overcomes some of the drawbacks of DPR [5]. However, this method also has limitations, such as higher radiation doses ranging between 19–368 mSv and DPR (2–9 mSv), particularly high levels of scattering and noise [6,7,8].

Many research groups have compared the diagnostic accuracy of DPR and CBCT. The meta-analysis of the in-vitro studies confirmed the ability of CBCT to discriminate a periapical pathosis from the healthy tissue being superior to that of DPR [9]. However, there is still lack of high-quality clinical evidence to demonstrate the diagnostic efficacy of CBCT relative to digital periapical radiography, mainly due to the absence of reference standard [10]. It has been suggested that the diagnostic accuracy of the radiographic images may depend on various diagnostic parameters, and in particular, on the selected threshold of the periapical disease definition [11]. Indeed, radiographic visualisation of periapical lesions may be influenced by individual variations of the anatomical structures, such as periodontal ligament space width or thickness of the bone [4].

Most of the previous reports focused on estimating various anatomical or treatment-related parameters performed using DPR and CBCT to demonstrate the potential superiority of one of the methods [12,13,14,15]. However, the periapical lesion size was the main factor influencing the radiographic detection of apical radiolucency [16]. It is important to note that the variations in diagnostic accuracy of either DPR or CBCT were reported to be greater for small lesions. Apparently, both radiographic examinations could detect larger lesions equally [16,17].

In a prospective study design, only a few reports have been found in the literature that compared the long-term dynamics of the periapical bone destruction after endodontic treatment as observed by DPR and CBCT. Thus, Patel et al., reported a significant difference in the outcome diagnosis of single roots when the paired data comparison was performed [18]. On the contrary, Liang et al., reported similar percentages of the absence and reduction of the periapical lesions after the long-term follow-up when assessed by DPR and CBCT [19]. The authors argued that the difference in definitions of the radiolucency size used in the studies plays a role in interpreting the study results [19].

The purpose of this study was to compare the radiographical periapical changes in size around the root canals after endodontic treatment over two years of follow-up assessed by DPR and CBCT and to analyse disagreements between the estimates obtained by two radiographic methods around the same roots using DPR and CBCT.

## 2. Materials and Methods

### 2.1. Subjects and Study Design

All patients referred for treatment with a specialist of endodontics at Vilnius Implantology Centre clinic (Vilnius, Lithuania) from December 2016 to December 2018 were invited to participate in this prospective study clinical study, with 2 years of follow-up. The Ethics Committee approved the study protocol of Lithuania Biomedical studies (Protocol No 111; edition No BE-2-27; 2016 12 20).

Of the total of 140 invited patients, 128 individuals with at least one tooth presented with radiographically detected periapical changes associated with necrotic pulp or root-filled teeth with radiographically determined signs of post-treatment endodontic disease. The signs were as follows: inadequate obturation length, homogeneity and not treated/missed root canals, presence of complications/failures, and requiring root canal retreatment were included in this study. Informed verbal and signed written consent was obtained from all participants before enrolment. The mean age of the study participants was 46 (SD 12.3) years, ranging between 18 and 70 years. The details of the inclusion and exclusion criteria and the study design have been reported previously [15,20].

Briefly, the exclusion criteria were: pregnant women, immunosuppressed patients, patients with symptoms of acute apical periodontitis, presenting with un-restorable teeth (e.g., deep carious lesions, coronal cracks, root fracture) and with probing depths >5 mm around the marginal bone.

### 2.2. Examinations of the Patients

All patients were examined clinically and radiographically (DPR and CBCT) after 2 years of endodontic treatment. Before the examination, every patient obtained a case history (presence of clinical symptoms such as pain, swelling, an abnormal bite, and time of previous treatment). The clinical examinations were performed by one examiner (JG). They included standard tests such as percussion, palpation, assessment of the coronal seal, presence of sinus tracts, tooth mobility, and periodontal probing depth.

Radiographic examinations were based on DPR and CBCT images assessment. A total of 162 DPR and 128 CBCT images of 176 teeth were obtained from 128 patients. Straight projection DPR images (Kodak, Carestream Dental LLC, Atlanta, GA, USA) were obtained using an intraoral sensor with the exposure parameters—60 kV, 7 mA and 0.3 s [21]. The CBCT images were obtained with an i-CAT scanner (Imaging Sciences International Inc., Hatfield, PA, USA); the exposure parameters were: 84 kV, 5 mA, 0.3 mm voxel resolution. The baseline CBCT images were performed for treatment planning purposes no earlier than one month prior to treatment [20]. The baseline CBCT images (360—rotation) were already available after the general diagnostic and treatment plan proposed by the specialists in prosthetics and implantology. The follow-up CBCT images were obtained 2 years after the endodontic treatment. Thus, the 180-rotation of the respective area of interest (maxillary or mandibular arch) was performed to minimise the radiation dose to the patients.

The DPR images were viewed as an original Kodak dental imaging presentation (Apple, Cupertino, CA, USA). The CBCT images were viewed using the original viewing program from i-CAT viewing software (Imaging Sciences International, Inc., Hatfield, PA, USA). All the obtained images were examined on the 27-inch flat-panel display computer screen (Apple, iMac, Cupertino, CA, USA) with a pixel resolution of 2.560 × 1.440 under a dimmed ambient light of less than 50 lux without time restrictions for the evaluations. The filters were set to normal, and only brightness and contrast were adjusted.

### 2.3. Analysis of Radiographic Images

All the obtained baseline and follow-up DPR and CBCT images were coded, mixed and evaluated by the principal examiner (JG) only, without knowing the clinical outcomes. To ensure the reliability of the measurements, training and calibration of this examiner with two other experienced examiners were performed prior to the study, using examples of both techniques. Cohen’s kappa was calculated to assess the intra-observer agreement, and the Fleiss kappa reflected the inter-examiner agreement. The inter-and intra-observer agreement was considered very good [15].

### 2.4. Treatment Procedures

Following the standardised protocol, the same qualified endodontist performed root canal treatment and retreatment procedures [20]. The root canals were prepared using sterile, single-use endodontic Flexofiles, Pathfiles, and ProTaper nickel-titanium (NiTi) rotary instruments (Dentsply Sirona, Ballaigues, Switzerland) under local anaesthesia with 1.7 mL of 4% articaine hydrochloride containing epinephrine hydrochloride (1:1,000,000) (Septodont, Saint-Maur-des-Fossés, France), and with intraoperative isolation using rubber dam system (Swedish Dental Supplies AB). The root canals were gently irrigated with 2 mL of 2.25% sodium hypochlorite (NaOCl) (Chloraxid-solution; PPH Cerkamed) between the instrumentations. For the final irrigation, 17% ethylenediaminetetraacetic acid (EDTA) (ENDO-Solution; PPH Cerkamed) solution was used for 1–2 min. NaOCl and EDTA irrigants were energised with a size 25 Endo-activator (Dentsply Sirona, USA) for 1 min. The canals were then dried with paper points and filled with gutta-percha and AHPlus sealer (Denstply Maillefer, Ballaigues, Switzerland) using a warm vertical compaction technique. The endodontically treated teeth were restored with permanent glass ionomer cores (GC Fuji IX) or with composite resin (Denstply Sirona, Charlotte, NC, USA), depending on the referring practitioner’s preference. A dental operating microscope (Carl Zeiss, Jena, Germany) with a medium magnification of 8× was used during the treatment procedures. The endodontic treatment procedures were completed in one or two visits, and the permanent restorations were performed within 1 month of root canal treatment.

### 2.5. The Diagnostic Parameters under Investigation

The periapical status of the teeth subjected to endodontic treatment was described following the radiographical diagnostic criteria by Venskutonis et al., For the present study purpose, only the size of radiolucency was analysed [22].

Thus, the size of radiolucency (S) was categorised according to the severity of periapical bone destruction as follows: S0—radiolucency does not exceed 2 times the width of the lateral periodontal ligament. S1—diameter of well-defined radiolucency up to 3 mm, S2—diameter of well-defined radiolucency 3–5 mm. S3—diameter of well-defined radiolucency >5 mm.

A CBCT and/or DPR detected radiolucency associated with the radiographic apex of the root, which was at least twice the width of the periodontal ligament space, was defined as periapical bone destruction.

The CBCT images that best confirmed the presence or absence of a radiolucent periapical lesion at least in two image planes (sagittal and/or coronal and/or axial) were selected.

The present study outcomes were classified:

positive treatment outcome, when the size of radiolucency around the endodontically treated canals reduced in size (represented by the transition events: S3→S2, S3→S1, S3→S0, S2→S1, S2→S0, S1→S0) and remained unchanged (S0↔S0, S1↔S1);

negative treatment outcome, when the size of radiolucency around the endodontically treated canals increased in size (represented by the transition events: S0→S1, S0→S2, S0→S3, S1→S2, S1→S3) and remained unchanged (S2↔S2, S3↔S3).

### 2.6. Statistical Analysis

The data were analysed using SPSS statistical software (version 27.0, SPSS Inc., Chicago, IL, USA). Descriptive statistics were used to report the distribution of the periapical radiolucencies around the endodontically treated root canals at baseline and two years after the treatment as well as their size transitions over the follow-up period, estimated by DPR and CBCT. The site-specific comparison of the radiolucencies identified by two methods at baseline and at follow-up examination after 2 years was performed using the Z test. The score disagreements in different lesion severity categories (S0, S1, S2, S3) were compared using Pearson’s chi-square test. For the comparison, the baseline categories S2 and S3 were combined due to the low numbers of these radiolucencies. Accordingly, the categories S1, S2 and S3 were connected for the comparison analysis after two years of follow-up. The statistical significance level was assumed at *p* < 0.050. Moreover, the data about the site-specific transition events of the periapical radiolucency size over two years was performed by presenting a number (and percentage) of cases where transition from one size category to another was recorded differently by DPR and CBCT.

## 3. Results

The study sample comprised a total of 176 teeth that yielded 395 and 403 root canals (corresponding to the same number of periapical radiolucencies) observed in the DPR and CBCT images, respectively. The distribution of the sample concerning the number of root canals identified by two radiographic methods is shown in Table 1.

**Figure 1:**
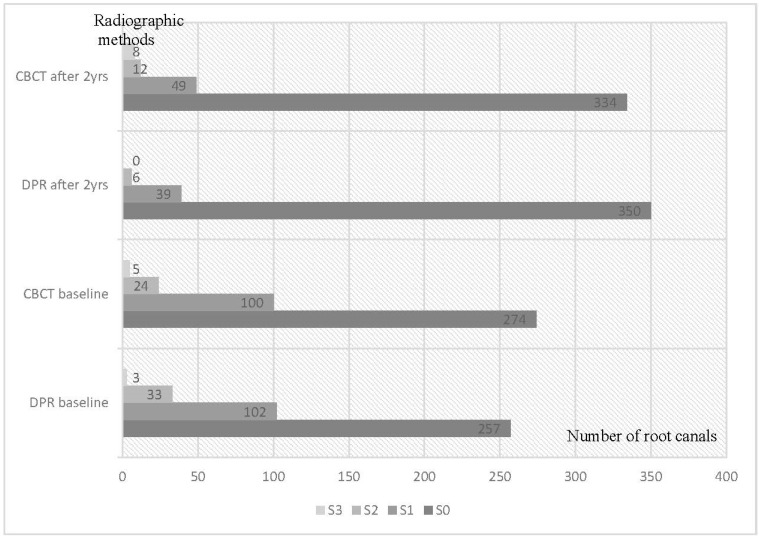
Overall distribution of scores for the size of radiolucency (S) detected by DPR and CBCT at baseline and 2 years of follow-up (n-root canal).

The overall distribution of the radiolucencies around the roots detected by DPR and CBCT at baseline and 2 years of follow-up is shown in Figure 1. The spectrum of the radiographically detected lesions looked very similar when the data obtained by CBCT and by DPR were compared. At baseline, the S0 scores accounted for two-thirds of the total number of all detected radiolucencies (65% in the DPR, and 68% in the CBCT images, respectively). The overall S0 scores increased two years after treatment, particularly the DPR estimates. Thus, the S0 scores comprised 89% and 83% of all detected radiolucencies at the follow-up examination by DPR and CBCT, respectively (Figure 1).

Regarding the most severe periapical lesions scored as S2 and S3, their baseline accounts comprised 9% and 7% of the total number of detected radiolucencies in the DPR and CBCT images, respectively. The percentages of S2 + S3 scores two years after the treatment were equal to 1.5% and 5% in the DPR and CBCT images, respectively.

Table 2 presents the non-matching scores of the periapical radiolucency size obtained by two radiographic methods (DPR and CBCT) when the site-specific analysis was performed at two examination sessions (baseline and follow-up, after two years). Thus, the non-matching scores at baseline comprised 18 and 20% of the total number of the detected radiolucencies by DPR and CBCT, respectively. There were more non-matching scores between the radiographic methods within the categories S1 and S2 + S3 than S0: at baseline; they comprised 17–33% of all detected radiolucencies within the category after two years, 62–95% of all detected radiolucencies within the category. Overall, the non-matching scores at baseline comprised 7%, 3% and 8% of the total observed radiolucencies in the categories S0, S1 and S2 + S3 by DPR and 11%, 7% and 1% by CBCT, respectively. At the follow-up examination, the non-matching scores comprised 11%, 6% and 1% of all detected lesions, in the categories S0, S1 and S2 + S3, by DPR and 7%, 8% and 5% by CBCT, respectively.

Analysis of the site-specific transition events of the periapical radiolucency size over two years as recorded by DPR and CBCT resulted in 130 cases where disagreements between two methods were identified. Of those, 84 cases (65% of all non-matching transition events) were related to the transitions between S0 and S1 (0→0, 0→1, 1→0, 1→1).

Table 3 indicates an existing disagreement in the treatment outcome assessment by CBCT and DPR after two years of follow-up. Thus, the CBCT-based evaluation resulted in negative treatment outcomes for 10 more root canals than the DPR-based result.

## 4. Discussion

The study results confirmed the existing variations in the detection of periapical radiolucencies when two radiographic methods (DPR and CBCT) were employed. The variations were already determined by the disagreement in the total number of identified root canals that accounted for 395 and 403 in the DPR and CBCT images, respectively. Table 1 indicates that the disagreements were generally more common in the mandibular teeth, with the tendency to identify more canals (and radiolucencies) in the CBCT images. These findings agree with the other studies [23,24]. Thus, Matherne et al., reported that when the root canal systems were investigated using DPR and CBCT images, the observer failed to identify at least 1 root canal in approximately 40% of teeth using DPR compared to CBCT [23]. Similarly, Sousa et al., demonstrated that in the premolars, CBCT identified 35% more root canals than DPR, mainly due to the poor capability of DPR to identify complex root canal configurations. This could be determined by the two-dimensional view of DPR radiographs and possible superimposition of the root canal features. In contrast, the three-dimensional view provided by CBCT images has been shown to have no problems with superimposition of the anatomical structures [24].

In the present study, the overall distribution pattern of the periapical lesions at baseline and after two years was similar when the DPR and CBCT results were compared. In general, the mild periapical radiolucencies (S0) accounted for two-thirds of the total number of all radiolucencies detected either by DPR or by CBCT at baseline. As expected, their number increased in two years after the endodontic treatment and comprised over 80% of all detected radiolucencies. It is important to note that the DPR estimates resulted in a 25% increase of S0 radiolucencies at the follow-up examination. In comparison, the CBCT estimates provided only 15%, thus indicating the less positive treatment outcome results when evaluated by means of CBCT.

Similarly, the two-year decrease in the percentage of severe periapical radiolucencies scored as S2 and S3 was only 2% based on the CBCT estimates, while 7.5% when the DPR images were assessed. The site-specific analysis of the treatment outcomes concerning the two-year transitions of the periapical radiolucencies detected by CBCT and DPR confirmed the notion that the CBCT-based evaluation resulted in more failed treatments than DPR. The present results can be explained by the 3D nature of the cone-beam computer tomography.

The most remarkable disagreement between the two radiographic methods in detecting periapical radiolucencies was observed within the lesion categories S2-S3. In particular, at the follow-up examination, the disagreement within these categories comprised up to 30% of the total amount of the recorded disagreements. The obtained results corroborate with the earlier findings of van der Borden et al. showing significant differences in the periapical lesion size when the volumetric CBCT and DPR data were compared [25]. The most likely explanation for such differences could be the compression of the 3-dimensional anatomy of the teeth and the surrounded anatomical structures into a 2-dimensional shadow in the DPR image, resulting in DPR’s inability to reveal the actual size and location of the periapical lesions [26,27]. As suggested previously, the overlying cortical and cancellous bones can mislead the observer of the DPR image and consequently, the size of radiolucency can be misinterpreted [28,29,30]. Venskutonis et al., proposed to include several anatomical parameters (lesion size, its location and relation to the root and the surrounding anatomical structures) in one index (COPI) deemed to evaluate the true nature of the periapical lesion and to assess the prognosis of root canal treatment [22]. Recently, we demonstrated that the lesion size, location, and relation to the root, when assessed in the CBCT images, correlate; thus, the treatment prognosis can be estimated using the lesion size only [20]. However, the applicability of COPI (as well as its reliability) was not validated in DPR images.

The analysis of the 2-year transitions of the periapical radiolucencies observed by CBCT and DPR showed that about 30% did not match (of the total of 395/403 radiolucencies detected by either method at baseline, 130 cases were recorded as non-matching transition events after two years). Interestingly, the majority (65%) of the non-matching transitions were associated with scores S0-S1. This indicates that the diagnostic accuracy of detecting small periapical lesions may be generally lower for both methods. Another possible reason for the relatively high number of non-matching events in the category of mild periapical lesions was the large proportion of such lesions in the total study sample. Such a limited variability of the periapical lesions in the study sample could be considered a weakness, limiting its representativeness. As shown earlier by Huumonen et al., the preoperatively healthy teeth are poorly suitable for follow-up studies of clinical variables [28]. Nevertheless, a prospective study design’s detailed analysis of the periapical changes after the endodontic treatment gave insight into the discriminatory abilities of different radiographic detection methods. As a potential study limitation a relatively large voxel size (0.3 mm) of the CBCT images could be considered. In endodontics, the size of the voxel varies from 0.1 to 0.4 mm although a smaller voxel size has been advocated to increase diagnostic accuracy [29]. However, there are no standardized protocols regarding the voxel size in CBCT for various diagnostic tasks in dentistry. The systematic review by Spin-Neto et al., indicates that images acquired in smaller voxel sizes, increase the radiation dose to the patient but might provide the same diagnostic outcome as lower resolution images [30]. Moreover, the use of a smaller voxel size to increase the image resolution can create some noise [31]. Therefore, the authors considered the threshold of 0.3 mm would be sufficient to detect a periapical lesion and to discriminate it from the healthy periodontium.

It is well recognised that treatment outcome evaluation should primarily be based on the clinical data regardless of the presence of radiolucencies in radiographic images [32]. The periapical radiolucency of asymptomatic endodontically treated teeth could represent scar tissue rather than periapical pathosis, leading to the potential dangers of overtreatment [33,34]. Moreover, one should remember that the periapical lesions may take longer to resolve when monitored by 3D imaging techniques [35].

## 5. Conclusions

Most remarkable disagreement between DPR and CBCT recordings was observed within the radiolucency categories S2 and S3. However, the diagnostic accuracy of both radiographic methods was questionable as it resulted in a high proportion of non-matching S0-S1 lesion transitions over 2 years.

## Figures and Tables

**Table 1:** Distribution of study sample by root canal type (N = 176 teeth).

Root Canal Types	Number of Root Canals Identified by Two Radiographic Methods, n	
	DPR	CBCT
Teeth with single root canal (incisors, canines, premolars, molars)	65	59
Teeth with two root canals (incisors premolars, molars)	60	72
Teeth with three root canals (premolars, molars)	162	156
Teeth with four root canals (molars)	108	116
Total of root canals, n	395	403

**Table 2:** Site-specific comparison of the non-matching scores of periapical radiolucencies detected by DPR and CBCT, concerning the severity of periapical radiolucency, at baseline and after two years.

	Diagnostic Categories of Periapical Radiolucency			
	S0	S1	S2 + S3	Total (n) (100%)
**Baseline examination** Number, n, and (%) of non-matching scores within diagnostic category, DPR/CBCT	27/44 * 11/16 (%)	32/30 * 31/30 (%)	12/5 * 33/17 (%)	71/79 18/20 (%)
Total, n, and (%) of detected radiolucencies by DPR/CBCT	257/274 65/68 (%)	102/100 26/25 (%)	36/29 9/7 (%)	395/403 100 (%)
**Follow-up examination** (after two years) Number, n, and (%) of non-matching scores within diagnostic category, DPR/CBCT	43/27 * 12/8 (%)	24/33 * 62/67 (%)	4/19 * 67/95 (%)	71/79 18/20 (%)
Total, n, and (%) of detected radiolucencies by DPR/CBCT	350/334 89/83 (%)	39/49 10/12 (%)	6/20 1/5 (%)	395/403 100 (%)

* Statistically significant differences between the numbers of non-matching scores within the category S0 as compared to S1, and S2 + S3, Z test, *p* < 0.050. After two years, the significant difference was reached when S1, S2 + S3 categories were combined.

**Table 3:** Treatment outcome evaluation concerning two-year transitions of the periapical radiolucencies detected by CBCT and by DPR.

	Negative Treatment Outcome CBCT/DPR (403/395) ^a^		Positive Treatment Outcome CBCT/DPR (403/395) ^a^	
	Changed to worse (S0→S1, S0→S2, S0→S3, S1→S2, S1→S3)	Remained unchanged (S2↔S2, S3↔S3)	Remained unchanged (S0↔S0, S1↔S1)	Changed to better (S3→S2, S3→S1, S3→S0, S2→S1, S2→S0, S1→S0)
N, number of transition events	15/11	9/3	299/275	80/106
Total (n)	24/14		379/381	

^a^ Total number of root canals identified by CBCT and DPR.

## Data Availability

The datasets used and/or analysed during the current study are available from the corresponding author on reasonable request.

## References

[1] Leonardi Dutra K., Haas L., Porporatti A.L., Flores-Mir C., Santos J.N., Mezzomo L.A., Correa M., Canto G.D. (2016). Diagnostic Accuracy of Cone-beam Computed Tomography and Conventional Radiography on Apical Periodontitis: A Systematic Review and Meta-analysis. J. Endod..

[2] Patel S., Patel R., Foschi F., Mannocci F. (2019). The Impact of Different Diagnostic Imaging Modalities on the Evaluation of Root Canal Anatomy and Endodontic Residents’ Stress Levels: A Clinical Study. J. Endod..

[3] Kanagasingam S., Lim C.X., Yong C.P., Mannocci F., Patel S. (2017). Diagnostic accuracy of periapical radiography and cone beam computed tomography in detecting apical periodontitis using histopathological findings as a reference standard. Int. Endod. J..

[4] Patel S., Brown J., Pimentel T., Kelly R.D., Abella F., Durack C. (2019). Cone beam computed tomography in Endodontics—A review of the literature. Int. Endod. J..

[5] Patel S., Dawood A., Mannocci F., Wilson R., Pitt Ford T. (2009). Detection of periapical bone defects in human jaws using cone beam computed tomography and intraoral radiography. Int. Endod. J..

[6] Stavropoulos A., Wenzel A. (2007). Accuracy of cone beam dental CT, intraoral digital and conventional film radiography for the detection of periapical lesions: An ex vivo study in pig jaws. Clin. Oral Investig..

[7] Keerthana G., Singh N., Yadav R., Duhan J., Tewari S., Gupta A., Sangwan P., Mittal S. (2021). Comparative analysis of the accuracy of periapical radiography and cone-beam computed tomography for diagnosing complex endodontic pathoses using a gold standard reference—A prospective clinical study. Int. Endod. J..

[8] Pauwels R., Beinsberger J., Collaert B., Theodorakou C., Rogers J., Walker A., Cockmartin L., Bosmans H., Jacobs R., Bogaerts R. (2012). Effective dose range for dental cone beam computed tomography scanners. Eur. J. Radiol..

[9] Pope O., Sathorn C., Parashos P. (2014). A comparative investigation of cone-beam computed tomography and periapical radiography in the diagnosis of a healthy periapex. J. Endod..

[10] Tsai P., Torabinejad M., Rice D., Azevedo B. (2012). Accuracy of cone-beam computed tomography and periapical radiography in detecting small periapical lesions. J. Endod..

[11] Petersson A., Axelsson S., Davidson T., Frisk F., Hakeberg M., Kvist T., Norlund A., Mejàre I., Portenier I., Sandberg H. (2012). Radiological diagnosis of periapical bone tissue lesions in endodontics: A systematic review. Int. Endod. J..

[12] Yapp K.E., Brennan P., Ekpo E. (2021). Endodontic disease detection: Digital periapical radiography versus cone-beam computed tomography-a systematic review. J. Med. Imaging.

[13] Sullivan J.E., di Fiore P.M., Koerber A. (2000). Radiovisiography in the detection of apical peri-odontitis. J. Endod..

[14] Paurazas S.B., Geist J.R., Pink F.E., Hoen M.M., Steiman H.R. (2000). Comparison of diagnostic accuracy of digital imaging by using CCD and CMOS-APS sensors with E-speed film in the detection of periapical bony lesions. Oral Surg. Oral Med. Oral Pathol. Oral Radiol. Endod..

[15] Gudac J., Hellén-Halme K., Venskutonis T., Puisys A., Machiulskiene V. (2020). Comparison of Selected Anatomical and Treatment-related Diagnostic Parameters Estimated by Cone-Beam Computed Tomography and Digital Periapical Radiography in Teeth with Apical Periodontitis. J. Oral Maxillofac. Res..

[16] Kruse C., Spin-Neto R., Evar Kraft D.C., Vaeth M., Kirkevang L.L. (2019). Diagnostic accuracy of cone beam computed tomography used for assessment of apical periodontitis: An ex vivo histopathological study on human cadavers. Int. Endod. J..

[17] Restrepo-Restrepo F.A., Can~as-Jimenez S.J., Romero-Albarracın R.D., Villa-Machado P.A., Perez-Cano M.I., Tobon-Arroyave S.I. (2019). Prognosis of root canal treatment in teeth with preoperative apical periodontitis: A study with cone-beam computed tomography and digital periapical radiography. Int. Endod. J..

[18] Patel S., Wilson R., Dawood A., Foschi F., Mannocci F. (2012). The detection of periapical pathosis using digital periapical radiography and cone beam computed tomography—Part 2: A 1-year post-treatment follow-up. Int. Endod. J..

[19] Liang Y.H., Jiang L., Gao X.J., Shemesh H., Wesselink P.R., Wu M.K. (2014). Detection and measurement of artificial periapical lesions by cone-beam computed tomography. Int. Endod. J..

[20] Gudac J., Hellén-Halme K., Machiulskiene V. (2021). Prognostic validity of the Periapical and Endodontic Status Scale for the radiographically assessed 2-year treatment outcomes in teeth with apical periodontitis: A prospective clinical study. BMC Oral Health.

[21] Durack C., Patel S., Davies J., Wilson R., Mannocci F. (2011). Diagnostic accuracy of small volume cone beam computed tomography and intraoral periapical radiography for the detection of simulated external inflammatory root resorption. Int. Endod. J..

[22] Venskutonis T., Plotino G., Tocci L., Gambarini G., Maminskas J., Juodzbalys G. (2015). Periapical and endodontic status scale based on periapical bone lesions and endodontic treatment quality evaluation using cone-beam computed tomography. J. Endod..

[23] Matherne R.P., Angelopoulos C., Kulild J.C., Tira D. (2008). Use of cone-beam computed tomography to identify root canal systems in vitro. J. Endod..

[24] Sousa T.O., Haiter-Neto F., Nascimento E.H.L., Peroni L.V., Freitas D.Q., Hassan B. (2017). Diagnostic Accuracy of Periapical Radiography and Cone-beam Computed Tomography in Identifying Root Canal Configuration of Human Premolars. J. Endod..

[25] van der Borden W.G., Wang X., Wu M.K., Shemesh H. (2013). Area and 3-dimensional volumetric changes of periapical lesions after root canal treatments. J. Endod..

[26] Bender I.B., Seltzer S. (1961). Roentgenographic and direct observation of experimental lesions in bone: I. J. Am. Dent. Assoc..

[27] Huumonen S., Ørstavik D. (2002). Radiological aspects of apical periodontitis. Endod. Top..

[28] Huumonen S., Orstavik D. (2013). Radiographic follow-up of periapical status after endodontic treatment of teeth with and without apical periodontitis. Clin. Oral Investig..

[29] Maret D., Peters A.O., Galibourg A., Dumoncel J., Esclassan R., Kahn J.-L., Sixou M., Telmon N. (2014). Comparison of the accuracy of 3-dimensional cone-beam computed tomography and micro-computed tomography reconstructions by using different voxel sizes. J. Endod..

[30] Spin-Neto R., Gotfredsen E., Wenzel A. (2013). Impact of voxel size variation on CBCT-based diagnostic outcome in dentistry: A systematic review. J. Digit. Imaging.

[31] Bechara B.B., McMahan A.C., Moore W.S., Noujeim M., Geha H., Teixeira F.B. (2012). Contrast-to-noise ratio difference in small field of view cone beam computed tomography machines. J. Oral Sci..

[32] Imura N., Pinheiro E.T., Gomes B.P., Zaia A.A., Ferraz C.C., Souza-Filho F.J. (2007). The outcome of endodontic treatment: A retrospective study of 2000 cases performed by a specialist. J. Endod..

[33] Liapatas S., Nakou M., Rontogianni D. (2003). Inflammatory infiltrate of chronic periradicular lesions: An immunohistochemical study. Int. Endod. J..

[34] Chang L., Umorin M., Augsburger R.A., Glickman G.N., Jalali P. (2020). Periradicular Lesions in Cancellous Bone Can Be Detected Radiographically. J. Endod..

[35] Garcia de Paula-Silva F.W., Hassan B., Bezerra da Silva L.A., Leonardo M.R., Wu M.K. (2009). Outcome of root canal treatment in dogs determined by periapical radiography and cone-beam computed tomography scans. J. Endod..

